# Serum Interleukin-23 in Polish Patients with Systemic Lupus Erythematosus: Association with Lupus Nephritis, Obesity, and Peripheral Vascular Disease

**DOI:** 10.1155/2017/9401432

**Published:** 2017-12-21

**Authors:** Katarzyna Fischer, Hanna Przepiera-Będzak, Marcin Sawicki, Anna Walecka, Iwona Brzosko, Marek Brzosko

**Affiliations:** ^1^Independent Laboratory for Rheumatologic Diagnostics, Pomeranian Medical University in Szczecin, Unii Lubelskiej 1, 71-252 Szczecin, Poland; ^2^Department of Rheumatology, Internal Medicine and Geriatrics, Pomeranian Medical University in Szczecin, Unii Lubelskiej 1, 71-252 Szczecin, Poland; ^3^Department of Imaging Diagnostics and Interventional Radiology, Pomeranian Medical University in Szczecin, Unii Lubelskiej 1, 71-252 Szczecin, Poland

## Abstract

**Objectives:**

To analyze the correlation between the serum concentration of interleukin- (IL-) 23 and atherosclerotic changes, traditional atherosclerotic risk factors, the autoantibody profile, and involvement of selected organs in systemic lupus erythematosus (SLE) patients.

**Patients and Methods:**

We studied 94 SLE patients and 27 controls. We analyzed the IL-23 serum concentration, autoantibodies, carotid intima-media thickness and atherosclerotic plaque, the ankle-brachial index, atherosclerotic risk factors, and organ manifestations.

**Results:**

Concentrations of IL-23 significantly differed between SLE patients and the controls (*p* = 0.0015). On the basis of multivariate stepwise analysis, we revealed that high levels of IL-23 were associated with atherosclerotic plaque in common femoral arteries (OR = 12.67; 95% CI: 1.41–113.84), lupus nephritis (OR = 3.69; 95% CI: 1.16–12.22), and obesity (OR = 4.21; 95% CI: 1.40–12.67). Autoantibodies related to IL-23 were anti-phosphatidylethanolamine antibodies (OR = 11.06; 95% CI: 1.24–98.65) and anti-SS-B/La antibodies (OR = 15.43; 95% CI: 1.73–137.25).

**Conclusions:**

IL-23 may be involved in lupus nephritis pathogenesis. Through its association with obesity and selected antiphospholipid antibodies, IL-23 might promote a hypercoagulable state contributing to atherothrombosis development in SLE patients.

## 1. Introduction

Systemic lupus erythematosus (SLE) is a chronic, inflammatory, multisystem autoimmune disease characterized by an irreversible break in immunologic self-tolerance and successive immune-mediated tissue damage [1]. A remarkable feature of the disease is the clinical heterogeneity, which indicates variations in underlying etiologic factors. The multifactorial etiology of SLE includes genetic susceptibility and hormonal and environmental triggers [2, 3]. Defective function of T cells and overactivation of B cells as well as defective clearance of apoptotic debris cause the production of autoantibodies, activation of complement, formation and deposition of immune complexes, and, consequently, tissue and organ damage [4, 5]. The crucial role in SLE pathogenesis plays innate and adaptive immune dysregulation, and it has been confirmed that certain cytokines are closely linked to SLE pathogenesis [6].

Interleukin- (IL-) 23 belongs to the IL-12 family cytokines and consists of two subunits, p19 and p40 [7]. IL-23 is produced by macrophages, dendritic cells, keratinocytes, and other antigen-presenting cells and through its interaction with the IL-23 receptor plays a central role in inflammation including the induction of Th17 cells [8, 9]. The IL-23-IL-17 axis is emerging as a critical regulatory system that bridges the innate and adaptive arms of the immune system and plays a critical role in development of autoimmune inflammatory diseases [10]. In fact, there are reports showing increased serum levels of IL-23 in systemic sclerosis [11], rheumatoid arthritis [12], primary antiphospholipid syndrome (PAPS) [13], spondyloarthropathies [14, 15], and inflammatory bowel diseases [16]. IL-23 has also been implicated in SLE [17–20], atherosclerosis [21, 22], and obesity [23].

However, to our knowledge, no complex data are available considering the additional relationship between serum concentrations of IL-23, atherosclerosis, and atherosclerotic risk factors in SLE patients.

## 2. Objectives

The aim of this study was to evaluate levels of IL-23 and their association with atherosclerotic changes, traditional atherosclerosis risk factors, disease characteristics including autoantibody profiles, and the involvement of selected organs in SLE patients.

## 3. Materials and Methods

This study was approved by the Local Ethics Committee of Pomeranian Medical University in Szczecin. Informed consent was obtained from all patients.

All patients were Caucasian. We studied 94 SLE patients and 27 healthy volunteers as the controls. The diagnosis of SLE was made according to the 1997 American College of Rheumatology Revised Criteria for Classification of Systemic Lupus Erythematosus [24].

The following data were recorded: age, gender, disease duration, activity of the disease according to Systemic Lupus Erythematosus Activity Index (SLEDAI) [1], antiphospholipid syndrome (APS) [25], lupus nephritis, cerebrovascular manifestations (transient ischemic attacks (TIA), stroke) and cardiovascular manifestations (coronary artery disease (CAD), myocardial infarction (MI)), Raynaud's phenomenon, and vasculitis.

All SLE patients and matched controls underwent noninvasive imaging investigations in the Department of Diagnostic Imaging and Interventional Radiology Pomeranian Medical University in Szczecin. All analyses were performed by the same experienced ultrasonographist with HDI 3500 (ATL) using a 5–12 MHz linear transducer. Carotid intima-media thickness (cIMT) measurements were performed with B-mode ultrasound in common carotid artery, bifurcation, and internal carotid artery on the right and left sides according to procedures previously described [26]. Due to the high variability of this parameter in populations [27, 28], the normal and pathological ranges of cIMT values were established on the basis of measurements in the controls [29]. The B-mode ultrasound was also used as a screening for atherosclerotic plaque presence in carotid and lower extremity arteries (the iliac, common femoral, deep femoral, superficial femoral, popliteal, and tibial arteries) [30]. The ankle-brachial index was assessed using Doppler ultrasonography and calculated as a ratio of systolic pressure measured in the posterior tibial and dorsal arteries of both feet to the systolic pressure in the brachial artery. Abnormal values were considered at ABI < 1.0 [31].

We assessed the presence of traditional risk factors of atherosclerosis: hypertension (systolic blood pressure ≥ 140 mmHg, diastolic pressure ≥ 90 mmHg), dyslipidemia (total cholesterol > 190.0 mg/dL, LDL cholesterol > 115.0 mg/dL, HDL cholesterol in males < 40.0 mg/dL and in females < 45.0 mg/dL, and triglycerides > 150.0 mg/dL), overweight and obesity based on body mass index (BMI) (overweight when BMI was 25 to <30, obesity when BMI was ≥30), diabetes, smoking habits, oral contraceptive use, and positive family history of cardiovascular diseases.

Blood was taken for the assessment of the erythrocyte sedimentation rate (ESR) (Westergren method) and C-reactive protein (CRP) (turbidimetric nephelometry), uric acid (modified Trinder assay based on the methods of Trivedi and Kabasakalian), homocysteine (fluorescent polarization immunoassay), total cholesterol (enzymatic, based on the formulation of Allain et al. and the modification of Roeschlau), direct LDL (low-density lipoproteins), direct HDL (high-density lipoprotein) cholesterol (enzymatic, colorimetric), direct triglycerides (enzymatic, colorimetric), glucose (hexokinase-mediated reaction), and fibrinogen (Clauss method).

The serologic diagnostics included the profile of autoantibodies determined with indirect immunofluorescence assay (IIFA), sandwich enzyme-linked immunosorbent assay (ELISA), and coagulation tests. IgG antinuclear antibodies (ANA) were assessed on the HEp-2 cell line contaminated by CVCL_0030 cervical adenocarcinoma human HeLa using the IIFA technique and monospecific tests performed with the ELISA method for detection of anti-double stranded DNA, anti-nucleosome, anti-Sm, anti-SS-A/Ro, anti-SS-B/La, anti-ribosomal P protein, anti-histone, and anti-U1-RNP antibodies (EUROIMMUN AG Medizinische Labordiagnostika tests, Lubeck, Germany). Antiphospholipid antibodies (aPLs) were determined with the ELISA method. The aPLs profile consisted of anticardiolipin (aCL) IgG, IgM, and IgA; anti-beta2-glycoprotein I IgG, IgM, and IgA (EUROIMMUN); antioxidized low-density lipoprotein IgG and IgM (IMTEC Immunodiagnostika, Berlin, Germany); antiprothrombin IgG, IgM, and IgA (AESKU.DIAGNOSTICS, Wendelsheim, Germany); antiphosphatidylserine (aPS) IgG and IgM (Demeditec Diagnostics, Kiel, Germany); antiphosphatidylethanolamine (aPE) IgG and IgM (The Binding Site, Birmingham, UK); and lupus anticoagulant (LA) performed with coagulation methods according to the International Society of Thrombosis and Haemostasis criteria [25]. The profile of anti-neutrophil cytoplasmic antibodies (ANCA) included screening IIFA for cytoplasmic (C-ANCA) and perinuclear (P-ANCA) and monospecific tests performed with the ELISA method for detection of anti-proteinase 3, anti-myeloperoxidase, anti-lactoferrin, anti-elastase, and anti-BPI antibodies (EUROIMMUN). The anti-endothelial cell antibodies were tested with human umbilical vein endothelial cells using the IIFA method (EUROIMMUN).

Serum was stored at −80°C until analysis for IL-23 using the sensitive ELISA method with the Quantikine Human IL-23 Immunoassay ELISA kit (minimum detectable dose less than 6.8 pg/mL). Kits were from R&D Systems, Minneapolis, USA. The system uses microplates coated with a monoclonal antibody and an enzyme-linked polyclonal antibody specific for IL-23. All analyses and calibrations were performed in duplicate and were read using BioTek PowerWave XS, BioTek Instruments, Winooski, USA.

All continuous variables were checked for equality distribution with Kolmogorov-Smirnov test. Data are described as the mean ± standard deviation and median (Q1, Q3). Comparison of continuous variables was performed by the Mann–Whitney *U* test. For categorical variables, differences were assessed by the logistic regression model and multivariate stepwise analysis. The logistic regression model and multivariate stepwise analysis probability (*P*) were assessed by a chi-square testing or Fisher's exact test. Results were shown as a *P* value, odds ratio (OR), and 95% confidence interval (95% CI) adjusted for sex and age. Findings were considered statistically significant at *P* < 0.05. Additionally, Bonferroni correction was used for related variables. All statistical analyses were performed with STATISTICA version 8.0, StatSoft Inc., Tulsa, OK, USA.

## 4. Results

The clinical and laboratory characteristics of the patients and healthy controls, including classical atherosclerotic risk factors and serologic profile, are presented in Tables 1, 2, 3, 4, and 5. The study group consisted of 82 women and 12 men suffering from SLE. The mean age was 44.5 years. The disease duration ranged from 1 to 30 years. The majority of patients presented low and medium SLE activity indices (52 and 33, resp.). The coexistence of APS was confirmed in 31, kidney involvement in 24, stroke in 10, and CAD in 11 SLE patients. The analysis of classical atherosclerosis risk factors revealed abnormal levels of total cholesterol in 52, LDL cholesterol in 40, HDL cholesterol in 5, and triglycerides in 25 patients with SLE. Hypertension was present in 37, overweight in 15, obesity in 23, diabetes in 11, smoking habits in 31, and positive family history of cardiovascular diseases in 4 SLE patients. The use of oral contraceptives confirmed 4 women suffering from SLE.  Table 5 shows the serological profile of SLE patients at the time the study was conducted. The frequency of ANA was 77.7%, which is lower than usually reported in SLE patients. However, the prevalence of ANA can change over time. In our patient group, the duration of the disease ranged from 1 to 30 years. Moreover, various immunosuppressive therapeutic schemes were applied in the patients and the majority of them went into remission which also might be the case of ANA disappearance in some of the SLE patients. The analysis of disease histories revealed that at the moment of diagnosis, all of them were positive for ANA.

**Figure 1:**
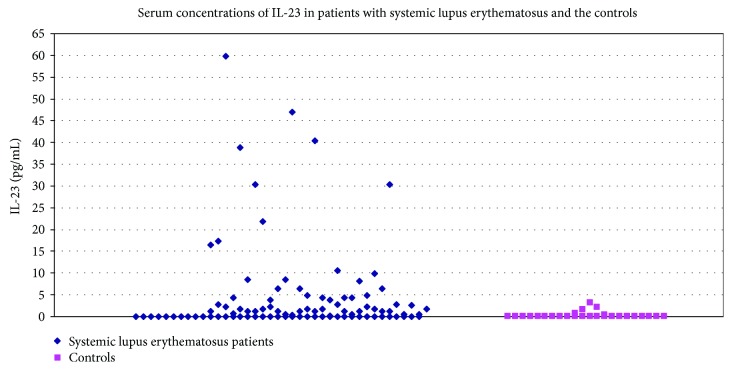
Serum concentrations of IL-23 in patients with systemic lupus erythematosus and the controls.

Serum levels of IL-23 were significantly higher in SLE patients than in the controls (*P* = 0.0015 after Bonferroni correction). The maximum level of IL-23 in the controls was 3.1 pg/mL and was established as the cut-off value (Table 6 and Figure 1).

The reference range of cIMT was established on the basis of the measurements in the controls. Intima-media thickness were considered moderately increased at values of 0.66–0.86 mm (51 SLE patients) and highly thickened at >0.86 mm (15 SLE patients). Values of ABI < 1.0 were found in 22 patients and <0.9 in five patients with SLE.

The OR for serum IL-23 > 3.1 pg/mL in SLE patients with the presence of atherosclerotic plaque in the right common femoral artery was 4.26 (95% CI 1.16–15.56), *P* = 0.029 (*P* = 0.058 after Bonferroni correction), and in the arteries of the lower extremities, it was 2.84 (95% CI 1.02–7.91), *P* = 0.046 (*P* = 0.092 after Bonferroni correction) (Table 7). The analysis of classical atherosclerotic risk factors revealed that the OR for the serum level of IL-23 > 3.1 pg/mL in SLE patients with obesity was 3.88 (95% CI 1.30–11.58), *P* = 0.015 (*P* = 0.015 after Bonferroni correction) (Table 7). There was no correlation between IL-23 and cIMT and ABI values as well as atherosclerotic plaque in the rest of the assessed arteries (data not shown). There was also no correlation between IL-23 and other classical atherosclerotic risk factors (data not shown). The OR for the serum level of IL-23 > 0.0 pg/mL in SLE patients with lupus nephritis was 3.18 (95% CI 1.06–9.54), *P* = 0.039 (*P* = 0.039 after Bonferroni correction) (Table 7). There was no correlation between IL-23 and other analyzed clinical complications in SLE patients (data not shown). Moreover, the OR for the serum level of IL-23 > 0.0 pg/mL in SLE patients with the presence of selected autoantibodies such as aPE IgG was 12.67 (95% CI 1.49–108.06), *P* = 0.020 (*P* = 0.100 after Bonferroni correction), aPE IgG or IgM was 4.61 (95% CI 1.19–17.88), *P* = 0.027 (*P* = 0.135 after Bonferroni correction), and aSS-B/La was 11.80 (95% CI 1.47–94.77), *P* = 0.020 (*P* = 0.020 after Bonferroni correction) (Table 7). The OR for the serum level of IL-23 > 0.0 pg/mL in SLE patients with aCL IgG was 2.26 (95% CI 0.9–5.71), *P* = 0.084 (*P* = 0.420 after Bonferroni correction), and with aPT IgG was 8.35 (95% CI 0.98–71.09), *P* = 0.052 (*P* = 0.260 after Bonferroni correction) (Table 7).

In the multivariate stepwise analysis, the OR for serum levels of IL-23 > 3.1 pg/mL in SLE patients with atherosclerotic plaque in the right common femoral artery was 4.27 (95% CI 1.08–16.89), *P* = 0.038, with obesity was 4.21 (95% CI 1.40–12.67), *P* = 0.011, and with the presence of aSS-B/La was 4.14 (95% CI 1.14–15.07), *P* = 0.031 (Table 8).

In the multivariate stepwise analysis, the OR for the serum level of IL-23 > 0.0 pg/mL in SLE patients with atherosclerotic plaque in the right common femoral artery was 12.67 (95% CI 1.41–113.84), *P* = 0.023, with obesity was 2.98 (95% CI 0.84–10.61), *P* = 0.091 (result at the border of statistical significance), with lupus nephritis was 3.69 (95% CI 1.11–12.22), *P* = 0.033, with the presence of aSS-B/La was 15.43 (95% CI 1.73–137.25), *P* = 0.014, and with aPE IgG was 11.06 (95% CI 1.24–98.65), *P* = 0.031 (Table 8).

There was no correlation between IL-23 and other analyzed laboratory and serologic characteristics in SLE patients (data not shown).

## 5. Discussion

The analysis of the role of selected proinflammatory and angiogenic cytokines in SpA patients [14, 15, 32, 33] showed higher levels of IL-23 in ankylosing spondylitis in comparison with the controls and revealed the association with selected clinical characteristics such as psoriatic onycholysis and the use of nonsteroid anti-inflammatory drugs [14, 15].

In addition, our initial reports [34] on atherosclerotic risk in SLE patients showed that risk factors for atherosclerosis in SLE differ from those observed in the general population. Chronic inflammation, the presence of selected autoantibodies (e.g., aPLs and AECA), and applied treatment (especially high cumulative dosage of glucocorticosteroids) are particularly important in atherosclerosis development. However, there is still the need to search for other pathomechanisms underlying this process.

In this study, we evaluated the relationship between serum levels of IL-23 and clinical and laboratory characteristics of SLE with the special focus on atherosclerosis and atherosclerotic risk factors. We found a significant correlation between high serum levels of IL-23 and atherosclerotic changes in the arteries of the lower extremity arteries. This finding stays in accordance with the reports on the IL-23 serum levels in patients affected by peripheral arterial disease (PAD) [22]. The authors analyzed 29 patients with advanced PAD: 28 had stage IV and one had stage III disease. All patients were qualified for lower extremity bypass (LEB) surgery using either autogenous vein or synthetic polytetrafluoroethylene (PTFE) graft material. Blood for measurement of IL-23 levels was taken at three points: 24 h before, 24 h after, and 5 days after the surgical intervention. IL-23 levels were higher at all three time points compared to the control group. Interestingly, 22 patients undergoing LEB with the autogenous vein had IL-23 levels greater than the seven patients who had synthetic PTFE graft material at all three time points. In our study, we did not find a significant correlation between serum levels of IL-23 and ABI values. This might be the result of the low number of patients with clinical manifestation of PAD. However, the number of SLE patients with ABI < 1.0 was 22, but significantly decreased values of this index <0.9 were found only in three patients, and among them, two confirmed intermittent claudication symptoms and only one underwent surgical intervention. In any case, the significant association between IL-23 levels and the presence of atherosclerotic plaque in lower extremity arteries, especially common femoral arteries, supports the hypothesis that IL-23 might be involved in PAD development in the course of SLE. Furthermore, there are data in the literature that address the possible role of the IL-23-IL-17 axis in carotid atherosclerosis [21] and in CAD [35].

Abbas et al. [21] showed that patients with carotid atherosclerosis had markedly raised plasma levels of IL-23 compared with healthy controls. The highest levels were present in patients with the most recent symptoms within the last two months. Moreover, IL-23 concentrations at follow-up after several years (mean 3.5) were associated with increased mortality mainly caused by cerebrovascular and cardiovascular events.

Khojasteh-Fard et al. [35] found a significant decrease of IL-23 gene expression in unstimulated peripheral blood lymphocytes of patients with CAD compared to those without CAD reinforcing the potential role of IL-23 in the complex mechanisms associated with the development of atherosclerosis. Data from patients who underwent diagnostic catheterization showed imbalances between Th17 and regulatory T cells and significantly higher levels of serum IL-23 along with IL-17 and IL-6 in those with acute MI and unstable angina compared with concentrations in patients with stable angina and chest pain syndrome [36]. Our study did not reveal a significant correlation of IL-23 serum levels and CAD, MI, stroke, and TIA as well as cIMT in SLE patients. To our knowledge, there are no available data in the literature concerning this issue. Some studies showed increased levels of IL-6 in SLE patients and the association with the burden of atherosclerosis [37]. Similarly, SLE patients with atherosclerosis showed increased levels of IL-6 and IL-17 compared with SLE alone and the controls [38]. The exact role of IL-23 and the IL-23-IL-17 axis in atherosclerosis and the development of complications in SLE patients still remains the issue that should be the matter of further studies.

In this study, we also analyzed the correlation between serum levels of IL-23 and classical atherosclerotic risk factors in SLE patients. We only found a significant association between IL-23 levels and obesity. Data from the study in 26 obese women and 20 lean volunteers showed significant increases of IL-23 levels as well as IL-17, leptin, and macrophage migration inhibitory factor (MIF) in obese subjects compared with the controls. Interestingly, there was no relationship between IL-12 and interferon *γ* and obesity. Moreover, the authors did not find the correlation between IL-23 and IL-17 and central obesity markers like BMI and waist circumference in obese women that suggests that the accumulation of IL-23-IL-17-producing cells in adipose tissue might not increase in proportion with the buildup of abdominal fat. It is also possible that blood mononuclear cells or some other cell type, rather than leukocytes infiltrating the adipose tissue, could be the main source of IL-17 and IL-23 in obese subjects. The role of the IL-23-IL-17 axis seems to be increased in obese women independently of proinflammatory mediators MIF and leptin [23]. These observations together with the data on the possible role of the IL-23-IL-17 proinflammatory cytokine axis in atherosclerosis development may indicate the important link between obesity and cardiovascular complications in SLE patients, and further research should be conducted to investigate this problem.

The association between SLE activity and clinical characteristics of the disease and IL-23 levels was also studied. We found significantly higher levels of IL-23 in SLE patients than in the controls, and there was also an important correlation of IL-23 serum concentrations and lupus nephritis, which is in accordance with other data. The relationship between the elevated serum levels of IL-23 and renal involvement in SLE patients was confirmed by several studies [39–41]. High serum IL-23 mRNA levels were also found in patients with SLE and renal disease [20]. Similarly, higher levels of serum IL-23 were found in SLE patients compared with the controls [17, 36, 42]. We did not confirm the relationship between SLE activity and other clinical characteristics of the disease. In the literature, contrary data are available on the association of IL-23 and SLE activity. Some reports showed a correlation between high levels of IL-23 and activity of the disease [18] as well as a relationship between IL-23 levels and selected manifestations like serositis and cutaneous involvement among patients with active disease [19]. Some reports presented discrepant results and did not show any association of serum IL-23 and SLE activity [12, 17, 36]. In our study, only nine patients had high activity of the disease according to the SLEDAI scale. The majority had low (52) or medium (33) activity, which may partly explain the lack of a relationship between IL-23 and activity of SLE.

Another interesting finding in our study was the significant correlation between serum IL-23 and selected autoantibodies including aPLs and aSS-B/La. Little data are available on the association between IL-23 and the serologic profile in SLE patients. Some studies did not find any correlation of IL-23 with autoantibodies typical for lupus like anti-double stranded DNA or anti-Sm [18, 19]. In fact, only the basic profile of autoantibodies was analyzed in these studies. One paper focused on IL-17 single-nucleotide polymorphisms and increased activity of the IL-23-IL-17 cytokine axis including serum measurements of IL-17 and IL-23, and tissue growth factor *β* in PAPS confirmed higher levels of IL-23 than that in controls. A significant correlation between IL-17 and IL-23 was reported. IL-17 was more frequently found in patients with deep vein thrombosis and thrombocytopenia. No significant associations between the analyzed cytokines and aPLs or clinical manifestations of PAPS were found [13]. In our previous observations in SLE patients with atherosclerotic changes, we found significant relationships between aPLs, secondary antiphospholipid syndrome, and atherosclerotic lesions [34, 43]. In this regard, the current finding is important to explain the possible mechanism of their involvement in atherosclerosis pathogenesis in SLE patients. Further studies are warranted to fully analyze the role of the IL-17-IL-23 cytokine axis in this phenomenon.

## 6. Conclusion

In conclusion, there are data, including ours, that support the involvement of IL-23 in SLE pathogenesis. Higher levels of IL-23 in patients compared with the controls and a significant correlation between IL-23 levels and selected clinical characteristics such as lupus nephritis were documented. The limitation of our study was that we analyzed only IL-23 serum levels, without the broad spectrum of proinflammatory cytokines. A relatively low number of patients presented cardiovascular and cerebrovascular manifestations. The majority of patients showed low activity of the disease. Even though we were able to confirm the interesting relationship between atherosclerotic lesions in the arteries of the lower extremities, obesity as well as selected aPLs and IL-23 levels in lupus patients also suggests the possible contribution of this cytokine in vascular involvement in the course of SLE, revealing new insights into atherothrombotic risk assessment in SLE patients. Further studies are necessary to fully elucidate this issue.

## Figures and Tables

**Table 1:** Clinical characteristics of systemic lupus erythematosus patients and healthy controls.

Assessed parameters	Systemic lupus erythematosus patients *n* = 94 Mean ± SD Median (Q1, Q3)	Healthy controls *n* = 27 Mean ± SD Median (Q1, Q3)
Age (years)	44.5 ± 13.5	43.6 ± 13.1
Sex	F = 82; M = 12	F = 21; M = 6
Disease duration (years)	7.0 (4.0, 12.0)	—
SLEDAI		
Low, *n* (%)	52 (55.3%)	—
Medium, *n* (%)	33 (35.1%)	—
High, *n* (%)	9 (9.6%)	—
APS, *n* (%)	31 (33.3%)	—
Lupus nephritis, *n* (%)	24 (25.5%)	—
Cerebrovascular manifestations		
TIA, *n* (%)	2 (2.1%)	—
Stroke, *n* (%)	10 (10.6%)	—
Cardiovascular manifestations		
CAD, *n* (%)	11 (11.7%)	—
MI, *n* (%)	4 (4.3%)	—
Raynaud's phenomenon, *n* (%)	25 (26.6%)	—
Vasculitis, *n* (%)	14 (14.9%)	—

SLEDAI: systemic lupus erythematosus disease activity index; APS: antiphospholipid syndrome; TIA: transient ischemic attacks; CAD: coronary artery disease; MI: myocardial infarction.

**Table 2:** Carotid intima-media thickness, ankle-brachial index, and atherosclerotic plaque in systemic lupus erythematosus patients and healthy controls.

Assessed parameters	Systemic lupus erythematosus patients *n* = 94 Median (Q1, Q3)	Healthy controls *n* = 27 Median (Q1, Q3)
cIMT (mm)	0.70 (0.65, 0.80)	0.60 (0.60, 0.70)
ABI right	1.08 (1.03, 1.16)	1.12 (1.06, 1.24)
ABI left	1.08 (1.10, 1.17)	1.15 (1.08, 1.21)
Plaques, *n* (%)		
cca, *n* (%)	3 (3.2%)	—
Bulb, *n* (%)	19 (20.2%)	—
ica, *n* (%)	2 (2.1%)	—
Carotid arteries, *n* (%)	22 (23.4%)	—
iliaca, *n* (%)	8 (8.5%)	—
cfa, *n* (%)	18 (19.1%)	—
dfa, *n* (%)	1 (1.1%)	—
sfa, *n* (%)	9 (9.6%)	—
popla, *n* (%)	4 (4.3%)	—
pta, *n* (%)	1 (1.1%)	—
Lower extremity arteries, *n* (%)	27 (28.7%)	—

cIMT: carotid intima-media thickness; ABI: ankle-brachial index; cca: common carotid arteries; ica: internal carotid arteries; iliaca: iliac arteries; cfa: common femoral arteries; dfa: deep femoral arteries; sfa: superficial femoral arteries; popla: popliteal arteries; pta: posterior tibial arteries.

**Table 3:** Laboratory characteristics of systemic lupus erythematosus patients and healthy controls.

Assessed parameters	Systemic lupus erythematosus patients *n* = 94 Median (Q1, Q3)	Healthy controls *n* = 27 Median (Q1, Q3)
IL-23 (pg/mL)	1.20 (0.0, 3.80)	0.0 (0.0, 0.0)
CRP (mg/L)	2.6 (1.2, 6.1)	0.0 (0.0, 1.0)
ESR (mm/h)	22.0 (12.0, 45.0)	10.0 (2.0, 16.0)
Fibrinogen (mg/dL)	316.0 (271.0, 374.5)	283.0 (246.5, 339.5)
Homocysteine (mol/L)	13.9 (11.0, 18.1)	6.6 (5.4, 8.1)
Uric acid (mg/dL)	4.6 (3.9, 5.9)	4.5 (3.5, 5.3)

IL-23: interleukin 23; CRP: C-reactive protein; ESR: erythrocyte sedimentation rate.

**Table 4:** Classical atherosclerotic risk factors in patients with systemic lupus erythematosus and healthy controls.

Risk factor	Systemic lupus erythematosus patients *n* = 94 Mean ± SD Median (Q1, Q3)	Healthy controls *n* = 27 Mean ± SD Median (Q1, Q3)
Total cholesterol (mg/dL)	218.5 ± 59.8	229.3 ± 41.3
LDL cholesterol (mg/dL)	129.1 ± 47.7	139.3 ± 34.3
HDL cholesterol (mg/dL)	58.9 ± 24.5	61.3 ± 9.9
Triglycerides (mg/dL)	150.0 ± 91.2	143.7 ± 73.3
Hypertension, *n* (%)	37 (39.3%)	3 (11.1%)
BMI	25.5 ± 4.9	24.5 ± 3.4
Smoking habits, *n* (%)	31 (33.0%)	12 (44.4%)
Diabetes, *n* (%)	11 (11.7%)	0 (0.0%)
Oral contraceptive use, *n* (%)	4/82 (4.9%)	5/21 (23.8%)
Family history of cardiovascular disease, *n* (%)	4 (4.3%)	4 (14.8%)

LDL: low-density lipoprotein; HDL: high-density lipoprotein; BMI: body mass index.

**Table 5:** Serological characteristics of systemic lupus erythematosus patients and healthy controls.

Autoantibody	Systemic lupus erythematosus patients *n* = 94 (%)	Healthy controls *n* = 27 (%)
Antinuclear antibodies IgG	73 (77.7%)	2 (7.4%)
Anti-double stranded DNA IgG	37 (39.4%)	0 (0.0%)
Anti-nucleosome IgG	30 (31.9%)	0 (0.0%)
Anti-Sm IgG	4 (4.3%)	0 (0.0%)
Anti-SS-A/Ro IgG	42 (44.7%)	0 (0.0%)
Anti-SS-B/La IgG	14 (14.9%)	0 (0.0%)
Anti-ribosomal P protein IgG	6 (6.4%)	0 (0.0%)
Anti-histone IgG	19 (20.2%)	0 (0.0%)
Anti-U1-RNP IgG	20 (21.3%)	0 (0.0%)
Anti-cardiolipin IgG	33 (35.1%)	0 (0.0%)
Anti-cardiolipin IgM	19 (20.2%)	1 (3.7%)
Anti-cardiolipin IgA	40 (42.6%)	1 (3.7%)
Anti-beta2-glycoprotein I IgG	7 (7.4%)	0 (0.0%)
Anti-beta2-glycoprotein I IgM	23 (24.5%)	1 (3.7%)
Anti-beta2-glycoprotein I IgA	23 (24.5%)	0 (0.0%)
Anti-oxidized low-density lipoprotein IgG	45 (47.9%)	0 (0.0%)
Anti-oxidized low-density lipoprotein IgM	68 (72.3%)	0 (0.0%)
Anti-prothrombin IgG	10 (10.6%)	0 (0.0%)
Anti-prothrombin IgM	12 (12.8%)	0 (0.0%)
Anti-prothrombin IgA	11 (11.7%)	0 (0.0%)
Anti-phosphatidylserine IgG	7 (7.4%)	0 (0.0%)
Anti-phosphatidylserine IgM	6 (6.4%)	1 (3.7%)
Anti-phosphatidylethanolamine IgG	12 (12.8%)	0 (0.0%)
Anti-phosphatidylethanolamine IgM	6 (6%)	0 (0.0%)
Lupus anticoagulant	15 (16.0%)	0 (0.0%)
Anti-neutrophil cytoplasmic antibodies IgG	39 (41.5%)	2 (7.4%)
Anti-proteinase 3 IgG	0 (0.0%)	0 (0.0%)
Anti-myeloperoxidase IgG	9 (9.6%)	0 (0.0%)
Anti-lactoferrin IgG	12 (12.8%)	0 (0.0%)
Anti-elastase IgG	9 (9.6%)	0 (0.0%)
Anti-BPI IgG	1 (1.1%)	0 (0.0%)
Anti-endothelial cell antibody IgG	42 (44.7%)	3 (11.1%)

BPI: bactericidal/permeability-increasing protein.

**Table 6:** Serum levels of IL-23 in patients with systemic lupus erythematosus and controls.

Serum level of IL-23 (pg/mL)	Systemic lupus erythematosus patients, *n* (%)	Controls, *n* (%)	OR (95% CI)	*P*	*P* ^#^
0.0	39 (41.4)	22 (81.8)	1.00		
0.01–3.10	30 (31.9)	5 (18.5)	3.38 (1.07–12.64)	0.0223	0.0669
>3.10	25 (26.6)	0 (0.0)	14.1 (3.55–60.48)	0.0005	0.0015

IL-23: interleukin-23; OR: odds ratio; 95% CI: 95% confidence interval; ^#^*P*: after Bonferroni correction. OR and *P* adjusted for sex and age.

**Table 7:** A logistic regression model of the OR of the increased serum level of IL-23 in systemic lupus erythematosus patients.

Covariates	Serum IL-23 > 0.0 pg/mL			Serum IL-23 > 3.1 pg/mL		
	OR (95% CI)	*P*	*P* ^#^	OR (95% CI)	*P*	*P* ^#^
Atherosclerotic plaque in cfa	10.12 (1.20–85.14)	0.033	0.066	4.26 (1.16–15.66)	0.029	0.058
Atherosclerotic plaque in lower extremity arteries in general	1.26 (0.48–3.32)	0.640	1.000	2.84 (1.02–7.91)	0.046	0.092
Obesity	3.83 (1.19–12.29)	0.024	0.024	3.88 (1.30–11.58)	0.015	0.015
Lupus nephritis	3.18 (1.06–9.54)	0.039	0.039	1.61 (0.52–4.97)	0.406	0.406
Anti-phosphatidylethanolamine IgG	12.67 (1.49–108.06)	0.020	0.100	0.71 (0.13–3.82)	0.687	1.000
Anti-phosphatidylethanolamine IgG or IgM	4.61 (1.19–17.88)	0.027	0.135	1.01 (0.28–3.62)	0.983	1.000
Anti-SS-B/La IgG	11.80 (1.47–94.77)	0.020	0.020	5.21 (1.53–17.71)	0.008	0.008
Anti-cardiolipin IgG	2.26 (0.90–5.71)	0.084	0.420	1.66 (0.61–4.49)	0.322	1.000
Anti-prothrombin IgG	8.35 (0.98–71.09)	0.052	0.260	0.64 (0.12–3.40)	0.598	1.000

IL-23: interleukin-23; cfa: common femoral arteries; OR: odds ratio; 95% CI: 95% confidence interval; ^#^*P*: after Bonferroni correction. OR and *P* adjusted for sex and age.

**Table 8:** The multivariate stepwise analysis for serum levels of IL-23 > 3.1 pg/mL and IL-23 > 0.0 pg/mL.

Covariates	Serum IL-23 > 3.1 pg/mL		Serum IL-23 > 0.0 pg/mL	
	OR (95% CI)	*P*	OR (95% CI)	*P*
Atherosclerotic plaque in cfa	4.27 (1.08–16.89)	0.038	12.67 (1.41–113.84)	0.023
Obesity	4.21 (1.40–12.67)	0.011	2.98 (0.84–10.61)	0.091
Anti-SS-B/La IgG	4.14 (1.14–15.07)	0.031	15.43 (1.73–137.25)	0.014
Lupus nephritis	NS	NS	3.69 (1.11–12.22)	0.033
Anti-phosphatidylethanolamine IgG	NS	NS	11.06 (1.24–98.65)	0.031

Cfa: common femoral artery; NS: nonsignificant. OR and *P* adjusted for sex and age.
